# COVID-19 VACCINE: THE CHALLENGE OF HERBAL MEDICINE COMMUNITY BELIEF IN A DEVELOPING COUNTRY – LETTER TO THE EDITOR

**DOI:** 10.21010/ajid.v15i2.1

**Published:** 2021-03-18

**Authors:** Firdian Makrufardi, Ade Saputri, Paulin Surya Phillabertha

**Affiliations:** 1* Department of Medicine, PKU Muhammadiyah Bantul General Hospital, Yogyakarta, Indonesia; 2 Department of Medicine, Majenang General Hospital, Central Java, Indonesia; 3 Department of Medicine, Nyi Ageng Serang General Hospital, Yogyakarta, Indonesia

**Keywords:** COVID-19, vaccine, herbal medicine, community, developing country

## Abstract

**Background:**

The first case of COVID-19 was officially confirmed by Indonesian government on the last March 2020, but the trend still shows no sign of decrease. In fact, traditional or herbal medicine have a big influence on people’s decisions about their health.

**Materials and Methods:**

This report describes the community belief in herbal medicine that provides immunity to COVID-19 infection.

**Results:**

In the early pandemic, there were so many false and misinformation about herbal that can cure COVID-19. They use mainly herbs and spices, eucalyptus oil, *arak Bali* as the alternative of COVID-19 remedies. People’s interest in using herbal also shown in the market influx of these things. In a condition where demand is higher than supply, the market ran out of stocks and the prices also sharply increased. Continuous research that uses herbal medicine as an alternative approach to COVID-19 treatment are still ongoing. Nevertheless, as of now, there is no concrete scientific evidence to support the use of traditional medicine in the treatment and management of COVID-19.

**Conclusion:**

These facts reflect that COVID-19 vaccine will face challenges in community. These challenges include misinformation, misleading information, cultures, and believes that potentially interfere the vaccination process. COVID-19 vaccine should get a place in peoples’ heart and mind thus can at least eliminate the pandemic.

## Introduction

The first case of COVID-19 was officially confirmed by Indonesian government in March 2020, but the trend now still shows no sign of decrease. Indonesia through Eijkman Institute still undergoes third clinical phase for the development of the vaccine (Ariawan and Jusril, 2020). In fact, the plan to provide vaccines to the public has many challenges, one of many factors that should be considered is the belief in herbal medicine that provides immunity to COVID-19 infection.

Clinical evidence from a range of studies of herbal medicine in the treatment of SARS coronavirus (SARS-CoV) has shown significant results and supported the idea that herbal medicine has a beneficial effect in the treatment and prevention of the epidemic disease. A Cochrane systematic review reported that herbal medicine combined with Western medicine may improve symptoms and quality of life in SARS-CoV patients (Liu *et al.*, 2012).

In COVID-19 cases, there is still no proof that herbal medicine can eliminate the cause of infection. Continuous research that uses herbal medicine as an alternative approach to COVID-19 treatment are still ongoing (Ang *et al.*, 2020). Nevertheless, as of now, there is no concrete scientific evidence to support the use of traditional medicine in the treatment and management of COVID-19 (Yang, 2020).

Deeply rooted as a culture in Indonesia, traditional or herbal medicine have a big influence on people’s decisions about their health. Driven by the claim that some herbal drugs or remedies can effectively treat COVID-19, some patients with flu symptoms who fear quarantine measures are likely to self-medicate with herbal remedies and avoid going to the hospital, thus delaying the proper diagnosis and treatment of the disease, and hampering the government’s testing, tracing, and quarantining efforts.

In the early pandemic, there were so many false and misinformation about herbal that can cure COVID-19. They use mainly herbs and spices, eucalyptus oil, *arak Bali* as the alternative of COVID-19 remedies. People’s interest in using herbal also shown in the market influx of these things. In a condition where demand is higher than supply, the market ran out of stocks and the prices also sharply increased (Indonesia C, 2020).

Herbal remedy seems to be the one that everyone turned into but a rushed judgement without sufficient scientific evidence should be cautioned. Even herbal medicine should be used rationally and correctly. People need reliable and accountable sources for this kind of information. The government should address this issue and explain it in the easiest way possible to make it understandable to every community level. These facts reflect that COVID-19 vaccine will face these same challenges in community. These challenges include: misinformation, misleading information, cultures, and believes that potentially interfere the vaccination process. COVID-19 vaccine should get a place in peoples’ heart and mind thus can at least eliminate the pandemic.

**List of abbreviation:**
COVID-19: : Coronavirus Disease 2019
SARS-CoV: : Severe Acute Respiratory Syndrome Coronavirus
